# Empirically derived guidance for social scientists to influence environmental policy

**DOI:** 10.1371/journal.pone.0171950

**Published:** 2017-03-09

**Authors:** Nadine Marshall, Neil Adger, Simon Attwood, Katrina Brown, Charles Crissman, Christopher Cvitanovic, Cassandra De Young, Margaret Gooch, Craig James, Sabine Jessen, Dave Johnson, Paul Marshall, Sarah Park, Dave Wachenfeld, Damian Wrigley

**Affiliations:** 1 CSIRO Land and Water, ATSIP Building, James Cook University, Townsville, QLD, Australia; 2 College of Environmental and Marine Sciences, James Cook University, Townsville, QLD, Australia; 3 Geography, College of Life and Environmental Sciences, University of Exeter, Rennes Drive Exeter, United Kingdom; 4 Biodiversity International, Bayan Lepas, Penang, Malaysia; 5 School of Environmental Sciences, University of East Anglia, Norwich Research Park, Norwich Norfolk, United Kingdom; 6 Environment and Sustainability Institute, University of Exeter, Penryn, Cornwall, United Kingdom; 7 World Fish Center, Bayan Lepas, Penang, Malaysia; 8 Centre for Marine Socioecology, University of Tasmania, Battery Point, Tasmania, Australia; 9 Food and Agriculture Organisation, Viale delle Terme di Caracalla Rome, Italy; 10 Great Barrier Reef Marine Park Authority, Townsville, Queensland, Australia; 11 CSIRO Land and Water, Canberra, Australia; 12 Canadian Parks and Wilderness Society and Simon Fraser University, Vancouver, Canada; 13 Department of the Environment, Canberra, Australia; 14 Centre for Biodiversity and Conservation Science, University of Queensland, St Lucia, Australia; 15 School of international Development, University of East Anglia, Norwich Research Park, Norwich Norfolk, United Kingdom; University of Sheffield, UNITED KINGDOM

## Abstract

Failure to stem trends of ecological disruption and associated loss of ecosystem services worldwide is partly due to the inadequate integration of the human dimension into environmental decision-making. Decision-makers need knowledge of the human dimension of resource systems and of the social consequences of decision-making if environmental management is to be effective and adaptive. Social scientists have a central role to play, but little guidance exists to help them influence decision-making processes. We distil 348 years of cumulative experience shared by 31 environmental experts across three continents into advice for social scientists seeking to increase their influence in the environmental policy arena. Results focus on the importance of process, engagement, empathy and acumen and reveal the importance of understanding and actively participating in policy processes through co-producing knowledge and building trust. The insights gained during this research might empower a science-driven cultural change in science-policy relations for the routine integration of the human dimension in environmental decision making; ultimately for an improved outlook for earth’s ecosystems and the billions of people that depend on them.

## Introduction

The future of natural resource systems is uncertain [1]. Rapid growth in economic activity is eroding the natural capital that underpins nearly every aspect of human existence and jeopardizes the prospects of operating within planetary boundaries [2–4]. In response, policy-makers are increasingly applying rules and regulations in an effort to meet society’s long term goals for the sustainable supply of ecosystem services [5]. However, restraints on human activities inevitably impose significant stress upon individuals, communities and industries, each of whom are expected to accept a decrease in environmental benefits and adapt. The resulting conflict, political turmoil, lack of compliance and transaction costs on authorities can seriously compromise conservation and sustainability goals [6–8]. Restraints still remain critical for our future well-being however they must be achievable, appropriate and acceptable. Despite decades of evidence on the importance of incorporating knowledge of people and their lives into the policy design and implementation process, policy-makers generally find it difficult to do so [9–12]. In fact, many resource protection policies fall short of, or directly contradict, what the available evidence suggests [13–16]. This omission is arguably one of the main factors contributing to the failure of environmental conservation and natural resource management worldwide [17,18].

The human dimension of environmental management is, of course, complex. There are competing and conflicting elements and values, and reconciling economic data with incommensurable, non-market and non-instrumental aspects is challenging [19,20]. A core mission of environmental social science is to identify pitfalls and possibilities associated with proposing and effecting interventions that conserve environmental integrity. Social scientists have models, theories and observations on how humans interact and relate to nature and its resources. Social scientists understand the complex consequences of policy and environmental change and the varying perspectives, values, experiences, motivations, aspirations, attachments, attitudes, behaviors, expectations, incentives, capacities and vulnerabilities of people dependent on natural resources including the conceptual, ethical and practical uncertainties involved with compensating people for the loss of nature [21–23][11] Squires 2009). The human dimension is their domain.

Given the scale of current environmental problems, integration of social science into environmental policy processes is urgent [24,25]. In fact, such calls have been commonplace for decades yet there is surprisingly little systematic scholarship to guide the process [26–28]. Saliency, legitimacy and credibility of science are known to be fundamental to the policy process [29]. Producing more knowledge does not, of itself, lead to greater policy influence [30,31]. Apparently, neither does educating policy makers [32–34]. Scientists are continually encouraged to reframe their work within relevant political contexts and engage more productively with policy-making processes [26,35,36] yet we see little evidence that such practices are occurring [37,38]. Our conclusion is that social scientists need a more comprehensive strategy for integrating social and behavioral evidence into environmental policy that they, themselves, can adopt. Hence, our aim is to identify constructive actions that could be taken by social scientists to increase the likelihood that they might influence environmental policy. Our approach is to learn from those whom have had direct and extensive experience operating at a high-level within the environmental science-policy nexus.

## Methods

CSIRO Social Science Human Research Ethics Committee approved this project (#063/14). Informed consent (oral and written) was obtained from each participant.

We interviewed 31 senior decision-makers, conservationists and environmental scientists for generic insights at the social science-policy nexus in Canada, Italy, Malaysia, UK, and Australia. Each organization in the study had developed environmental policy with social science input. The experts that participated in the study were selected for their diversity in experience across environmental issues, positions within the science-policy interface and across jurisdictions and styles. They were also reputed for their interest and/or effectiveness in integrating social science data into policy processes. In many instances, a key staff member was identified within each organization and asked to identify the most appropriate personnel with expertise at the science-policy nexus and that could provide exceptional advice. Twenty-seven participants had worked in a policy environment, nine had worked as a social scientist and five had worked as an ecologist.

### Survey design

A three-question qualitative survey was designed to elicit advice for policy influence. The first question was designed to focus research participants on the research problem at hand. We asked them to describe their experiences relating to the use of social science and its integration into environmental management and policies. Our second question was aimed towards gaining richer and constructive insights from their experience. We asked the experts to analyze or elaborate on each experience for key learnings. The third question was posed to convert their learnings into well-constructed advice that could be directly converted into a ‘tip’. Participants were asked to reflect on their learnings and provide advice to social scientists working in the field of environmental and natural resource management. The survey questions were:

1. Can you please describe your experience at the policy/social science interface? (probe for several examples and detail)
2. Let’s go through each example. Can you elaborate on what you think the key learnings might be–what were the main problems and issues for obtaining and integrating social science data into policy processes? Where was it easy?
3. Let’s now convert your learnings into advice for social scientists. How would you best inspire scientists to influence policy processes? (probe for advice for each example).

### Survey administration

We interviewed 31 senior decision-makers, conservationists and scientists across Canada (Canadian Parks and Wildlife Service, Vancouver, n = 1), Malaysia (WorldFish, Penang n = 6), Italy (Food and Agriculture Organisation, Rome, n = 12), UK (University of Exeter, Exeter and Falmouth, n = 2) and Australia (Commonwealth Scientific and Industrial Research Organization, Great Barrier Reef Marine Park Authority, and Department of the Environment, Townsville and Canberra, n = 2, 3, 6). All interviews were conducted by the first author as a face-to-face interview in or near the offices of each participant. Interviews were not recorded, but rather detailed notes were taken during the interviews, with a particular emphasis on capturing ‘advice’.

### Survey data analysis

Some participants were able to offer numerous pieces of advice to social scientists, whereas others preferred to focus on detailing just a few pieces of advice. In sum, we obtained a list of 128 pieces of advice from the notes taken, many of which were overlapping and sometimes contradictory. Each response was thematically coded by the first author using professional judgement, and manually clustered (in Word) for commonalities and similar meanings. Each cluster was tentatively titled to reflect the content within each cluster and refined amongst the co-authors. The content within each cluster was heavily edited for space efficiency by all authors but within keeping of the original intent of the information. A number of the clusters were merged together and some were relegated as less important according to the authors that had provided those comments, so that the final list of ‘tips’ was capped at ten. Our analysis is presented as a list of advice or tips for social scientists who wish to have greater relevance to, and impact on, environmental policy. Whilst generic, not all tips will be useful across all contexts. They are also not mutually exclusive but instead emphasize key themes that arose during the interviews.

## Results

### Acquire policy acumen

Decision-making is about making choices in the face of imperfect knowledge, risk, tight time-frames and complexity. Sometimes science is part of the process—sometimes not. To contribute, social scientists must develop an awareness of the policy world and understand that priorities can change quickly. There are formal avenues for decision-making and less formal ones, and scientists can access and support both. Scientists should know that knowledge that fits within pre-conceived value sets has more potential to be influential and that not all knowledge gaps are equally important to decision-makers. Scientists should recognize where scientific knowledge may actually enable or inform a decision and target their research explicitly here. Scientists might best acquire acumen of the constantly evolving policy space through developing relationships with policy makers, attending policy events and keeping abreast of policy processes and outputs.

### It’s all about process

Influencing decision-making is an art. It is about engagement and relationships and co-producing knowledge. Social scientists that set out to be part of the policy process from the beginning are more likely to be effective. The more time scientists spend with decision-makers the more likely decision-makers will assimilate information osmotically. Social scientists should consider engaging policy advisors or those decision-makers that are more likely to need social science knowledge and enhance their capacity to use social science such as through running a course or delivering a brief. Social scientists will need to be creative in how they become a trusted advisor and consider cooperative modes of conducting science and coproducing knowledge. Scientists could temporarily embed themselves in a policy section through secondments or as part of a collaborative project, or ask for formal introductions into the broader policy arena.

### Sit in their seat

Decision-making processes are frequently participative. Social scientists are more likely to be invited into the process if empathetic to the competing goals of policy. They should become aware of, and acknowledge, political and policy realities, as well as the complexities and challenges of decision-making and be explicit about their personal motivations and goals and how these align with the needs of the decision-maker. They should focus on the policy problem and help decision-makers to consider the risks, uncertainties and complexities and canvass policy options without bias towards their own work. Social scientists must develop trust. Developing trust is about enabling decision-makers to feel comfortable to open up, take appropriate risks and share vulnerabilities. It can be formed through trusting others, communicating well, developing a healthy working relationship.

### Be free to focus on strong science

Science should be directed towards the most pressing problems of the day, however social scientists will recognize the trade-offs with innovation in designing their science for policy. Social scientists should think about the contribution that they want to make with their science in broader terms and not be constrained by policy requirements. Policy-makers will often prefer to work with scientists that have already established themselves in their field. If the science is excellent and relevant, social scientists will gain a reputation for providing vision and insight into environmental problems, at which point social scientists can use their authority to simplify and generalize beyond the comfort zone of a conventional scientist.

### Engage, educate and enable

The relationship between scientists and the public is changing. In contemporary society citizens often express their beliefs in, and doubts of, science. The opinion of the voting public, the media and powerful, vested interests are often more influential to policy processes than science. Influencing these and helping the public understand socio-environmental issues can be an effective way to increase literacy about an environmental issue and its challenges and help ensure that public influence on policy aligns with the current state of the science. Social scientists are well placed to engage the public and should avoid using the language of neutrality when issues are not neutral. That is, scientists have a role in describing the political nuances of research findings. In developing messages, scientists should be compelling, clear, and authoritative, and should not hide behind the science (for example: numbers and probabilities can be confusing, imprecise and overly qualified). Too much detail can alienate an audience (detail should be left for publications). It is often more compelling to use comparisons and rankings such as highest, lowest, increasing and decreasing. Data in accessible forms such as infographics, narratives or scenarios are also more influential.

### Consider brokering

The messenger can be more influential than the message. For example, Pope Francis is likely to have greater influence on climate change mitigation than most scientists as a result of his role in society. While most scientists cannot aspire to this degree of social currency, some social scientists have successfully used NGOs, science advisors and key social identities to act as champions or activists for their science and for the environment. Knowledge brokering is emerging as a distinct specialization and can be effective as a way to influence policy. Brokers can be particularly effective if they are senior, sit in a policy department and can guide research. However social scientists need to be discerning about how best to use brokers. Scientists lean towards wanting to use brokers for efficiency and communication expertise; environmental managers often prefer to work directly with scientists that are perceived to be more passionate, technically savvy and authoritative with the public.

### Foresee opportunity

Sometimes it is not possible to influence policy processes once they are in train. This is when scientists need to identify where and when future opportunities might emerge. What is driving decisions today could shift tomorrow so an ability to respond quickly and positively will be crucial. A change in government, minister or senior bureaucrat, each represent a new policy-window. Formal and informal new networks offer other opportunities. Employing a political scientist can help identify other policy openings or prospects. Opportunities are often best met when scientists can give timely advice when needed.

### Integration is the new black

Approaching environmental issues through a systems understanding is critical to address complex dynamic relationships. Working across disciplines helps expose knowledge blind-spots, questions assumptions, exposes trade-offs and synergies and leads to better solutions. Social scientists need to team up with biophysical scientists to provide a shared perspective on policy advice. Social scientists are well placed to lead the integration of social science data with those of the biophysical sciences. They should do this by sharing knowledge, focusing on process, output and outcome, and acknowledging disciplinary differences in science approaches.

### Know thy strengths

Social scientists can guide policy if they are practical, hands-on and provide the necessary insights into the social dimension. They must recognize what they are personally good at and where their discipline excels, and offer these to the policy problem at hand. Multiple perspectives, competing values, complexity and problem framing are some challenges that social scientists are particularly adept at tackling, each of which are critical to developing acceptable policy options. Social scientists that help decision-makers to address the social aspects of environmental decision-making and can progress solutions can be indispensable.

### Validate and add value

Social scientists are often expected to bring about cultural and behavioural change even though this role may not quite meet the expectations of social scientists nor represent the spectrum of social science skills a social scientist possesses. Nevertheless, helping the public to understand the complexity in environmental decision-making and how an environmental decision was reached (using the latest scientific findings) provides an essential service to decision-makers. Social scientists that go ‘above and beyond’ what is required of them and add value to research by assisting in its facilitation or implementation, or by validating decisions within the public arena can achieve significant impact sooner.

## Discussion and conclusions

Without sound social science in the policy process, environmental decision-makers are condemned to repeat the mistakes of the past and demote science to merely charting the ongoing decline and degradation of the environment [39,40]. We argue that a relentless focus on informing policy through understanding and actively participating in policy processes as well as co-producing knowledge and building trust will yield dividends that lay the foundations for more resilient and adaptive environmental and sustainable decision-making.

The ten tips identified in this study are the distillation of a wealth of personal experiences from leading experts at the science-policy interface, and they align strongly with the convergence of a diverse body of research relating to science impact [27,41–45]. The advice, therefore, is likely to be more broadly useful to scientists than to those only within the social sciences. However, the right enabling conditions will be necessary if the tips are ever to be effectively implemented, regardless of the disciplinary orientation of the scientists [46,47]. Decision-makers will need to be receptive and more adventurous in the forms of knowledge they use.

We also note that nearly all of the tips require a serious time commitment on the part of the scientist and that this may go against the research goals of the scientist or their host institution. This tension is perhaps unfair to place on scientists. Given that policy influence requires this time commitment, institutional innovation will be required by research institutions to promote a culture whereby policy engagement activities are legitimized as core business and recognized and rewarded appropriately. This should include formally recognizing engagement and communication activities as a core component of a scientist’s role, and supporting these activities with both dedicated funding, time allocations and training. At the same time scientists should be rewarded for engagement and outreach activities alongside traditional metrics of science impact such as peer-reviewed.and employers of social scientists and other scientists will need to create the space and incentive structures for policy engagement processes [28,48,49]. We have seen the benefits of these ideas in practice. It is inevitable that social scientists will play an increasingly instrumental role in shaping policy and guiding environmental decisions. We hope that, within this era of dramatic change and unprecedented sustainability challenges, this paper can facilitate progress towards more equitable and effective decisions.

## References

[1] Clark WC, Kates RW, Corell R, Hall JM, Jaeger CC, et al (2001) Environment and development—Sustainability science. Science 292: 641–642. 11330321 10.1126/science.1059386

[2] Rockstrom J, Steffen W, Noone K, Persson A, Chapin FS, et al (2009) A safe operating space for humanity. Nature 461: 472–475. 10.1038/461472a 19779433

[3] Rockstrom J, Steffen W, Noone K, Persson A, Chapin FS, et al (2009) Planetary Boundaries: Exploring the Safe Operating Space for Humanity. Ecology and Society 14.

[4] Scheffer M, Carpenter S, Foley JA, Folke C, Walker B (2001) Catastrophic Shifts in Ecosystems. Nature 413: 591–596. 10.1038/35098000 11595939

[5] Game E, Schwartz MW, Knight AT (2015) Policy Relevant Conservation Science Conservation Letters 8: 309–311.

[6] Jones PJS (2006) Collective action problems posed by no-take zones. Marine Policy 30: 143–156.

[7] Kalfagianni A, Pattberg P (2013) Participation and inclusiveness in private rule-setting organizations: does it matter for effectiveness? Innovation-the European Journal of Social Science Research 26: 231–250.

[8] McClanahan T, Cinner J, M., Maiana J, Graham NAJ, Daw TM, et al (2008) Conservation Action in a Changing Climate. Conservation Letters 1: 53–59.

[9] Freudenberg WR, Gramling R (2002) Scientific Expertise and Natural Resource Decisions: Social Science Participation on Interdisciplinary Scientific Committees. Social Science Quarterly 83: 119–136.

[10] Mascia MB, Brosius JP, Dobson TA, Forbes BC, Horowitz LS, et al (2003) Conservation and the Social Sciences. Conservation Biology 17: 649–650.

[11] Mascia MB (2003) The Human Dimension of Coral Reef Marine Protected Areas: Recent Social Science Research and Its Policy Implications. Conservation Biology 17: 630–632.

[12] Young JC, Waylen KA, Sarkki S, Albon S, Bainbridge I, et al (2014) Improving the science-policy dialogue to meet the challenges of biodiversity conservation: having conversations rather than talking at one-another. Biodiversity and Conservation 23: 387–404.

[13] Adams WM, Sandbrook C (2013) Conservation, evidence and policy. Oryx 47: 329–335.

[14] Juntti M, Russel D, Turnpenny J (2009) Evidence, politics and power in public policy for the environment. Environmental Science & Policy 12: 207–215.

[15] Wilson KA, McBride MF, Bode M, Possingham HP (2006) Prioritizing global conservation efforts. Nature 440: 337–340. 10.1038/nature04366 16541073

[16] Young K, Ashby D, Boaz A, Grayson L (2002) Social Science and the Evidence-based Policy Movement. Social Policy & Society 1: 215–224.

[17] Endter-Wada J, Blahna D, Krannich R, Brunson M (1998) A framework for understanding social science contributions to ecosystem management. Ecological Applications 8: 891–904.

[18] Mcleod E, Szuster B, Hinkel J, Tompkins EL, Marshall N, et al (2015) Conservation organizations need to consider adaptive capacity: why local input matters. Conservation Letters 9.

[19] Adger WN, Barnett J, Chapin FS, Ellemor H (2011) This Must Be the Place: Underrepresentation of Identity and Meaning in Climate Change Decision-Making. Global Environmental Politics 11: 1-+.

[20] Varjopuro R, Gray T, Hatchard J, Rauschmayer F, Wittmer H (2008) Introduction: Interaction between environment and fisheries—The role of stakeholder participation. Marine Policy 32: 147–157.

[21] Kittinger JN, Finkbeiner EM, Glazier EW, Crowder LB (2012) Human Dimensions of Coral Reef Social-Ecological Systems. Ecology and Society 17.

[22] Lacey J, Howden M, C C, Dowd A (2015) Informed adaptation: Ethical considerations for adaptation researchers and decision-makers. Global and Environmental Change 32: 200–210.

[23] Squires D (2009) Opportunities in Social Science Research. Future of Fisheries Science in North America 31: 637–696.

[24] Kates RW, Clark WC, Corell R, Hall JM, Jaeger CC, et al (2001) Environment and development—Sustainability science. Science 292: 641–642. 11330321 10.1126/science.1059386

[25] Lubchenco J (1998) Entering the century of the environment: A new social contract for science. Science 279: 491–497.

[26] Fazey I, Evely AC, Reed MS, Stringer LC, Kruijsen J, et al (2013) Knowledge exchange: a review and research agenda for environmental management. Environmental Conservation 40: 19–36.

[27] Reed MS, Stringer LC, Fazey I, Evely AC, Kruijsen JHJ (2014) Five principles for the practice of knowledge exchange in environmental management. Journal of Environmental Management 146: 337–345. 10.1016/j.jenvman.2014.07.021 25194520

[28] Cvitanovic C, Hobday A, van Kerkhoff L, Wilson SK, Dobbs K, et al (2015) Improving knowledge exchange among scientists and decision-makers to facilitate the adaptive governance of marine resources: a review of knowledge and research needs. Ocean & Coastal Management.

[29] Cash DW, Clark WC, Alcock F, Dickson NM, Eckley N, et al (2003) Knowledge systems for sustainable development. Proceedings of the National Academy of Sciences of the United States of America 100: 8086–8091. 10.1073/pnas.1231332100 12777623 PMC166186

[30] Agrawal A, Ostrom E (2006) Political science and conservation biology: a dialog of the deaf. Conservation Biology 20: 681–682. 16909553 10.1111/j.1523-1739.2006.00468.x

[31] Rose DC (2015) The case for policy-relevant conservation science. Conservation Biology 29: 748–754. 10.1111/cobi.12444 25545991 PMC4510816

[32] Boyd I (2013) A standard for policy-relevant science. Nature 501: 159–160. 24032133 10.1038/501159a

[33] Fish RD (2011) Environmental decision making and an ecosystems approach: Some challenges from the perspective of social science. Progress in Physical Geography 35: 671–680.

[34] Sutherland WJ, Pullin AS, Dolman PM, Knight TM (2004) The need for evidence-based conservation. Trends in Ecology & Evolution 19: 305–308. 16701275 10.1016/j.tree.2004.03.018

[35] Sarewitz D, Pielke RA (2007) The neglected heart of science policy: reconciling supply of and demand for science. Environmental Science & Policy 10: 5–16.

[36] Symes D (1996) Fisheries Management and the Social Sciences: A Way Forward? Sociologia Ruralis 36: 146–151.

[37] Cvitanovic C, Wilson SK, Fulton CJ, Almany GR, Anderson P, et al (2013) Critical research needs for managing coral reef marine protected areas: Perspectives of academics and managers. Journal of Environmental Management 114: 84–91. 10.1016/j.jenvman.2012.10.051 23220604

[38] Cvitanovic C, Crimp S, Fleming A, Bell J, Howden M, et al (2016) Linking adaptation science to action to build food secure Pacific Island communities. Climate Risk Management 11: 53–62.

[39] Rudd MA, Beazley KF, Cooke SJ, Fleishman E, Lane DE, et al (2011) Generation of Priority Research Questions to Inform Conservation Policy and Management at a National Level. Conservation Biology 25: 476–484. 10.1111/j.1523-1739.2010.01625.x 21175828 PMC3108069

[40] Sarewitz D (2004) How science makes environmental controversies worse. Environmental Science & Policy 7: 385–403.

[41] Cook CN, Mascia MB, Schwartz MW, Possingham HP, Fuller RA (2013) Achieving Conservation Science that Bridges the Knowledge-Action Boundary. Conservation Biology 27: 669–678. 10.1111/cobi.12050 23574343 PMC3761186

[42] Fazey I, Bunse L, Msika J, Pinke M, Preedy K, et al (2014) Evaluating knowledge exchange in interdisciplinary and multi-stakeholder research. Global Environmental Change-Human and Policy Dimensions 25: 204–220.

[43] Gibbons P, Zammit C, Youngentob K (2008) Some practical suggestions for improving engagement between researchers and policy-makers in natural resource management. Ecological Management & Restoration 9: 182–186.

[44] Heck N, Stedman RC, Gaden M (2015) The integration of social science information into Great Lakes fishery management: Opportunities and challenges. Fisheries Research 167: 30–37.

[45] Roux DJ, Stirzaker RJ, Breen CM, Lefroy EC, Cresswell HP (2010) Framework for participative reflection on the accomplishment of transdisciplinary research programs. Environmental Science & Policy 13: 733–741.

[46] Cvitanovic C, Hobday AJ, van Kerkhoff L, Marshall NA (2015) Overcoming barriers to knowledge exchange for adaptive resource management; the perspectives of Australian marine scientists. Marine Policy 52: 38–44.

[47] van Kerkoff L (2002) Making a Difference: Science, Action and Integrated Environmental Research [PhD Thesis]. Canberra: Australian National University.

[48] Jacobson N, Butterill D, Goering P (2004) Organizational factors that influence university-based researchers' engagement in knowledge transfer activities. Science Communication 25: 246–259.

[49] Shanley P, Lopez C (2009) Out of the Loop: Why Research Rarely Reaches Policy Makers and the Public and What Can be Done. Biotropica 41: 535–544.

